# Effect of Covid-19 on households welfare in Afar Regional State, Ethiopia

**DOI:** 10.1007/s43621-022-00095-6

**Published:** 2022-08-08

**Authors:** Dagmawe Menelek Asfaw, Abdurhman Kedir Ali, Mohammed Adem Ali

**Affiliations:** Department of Economics, College of Business and Economics, Samara University, P.O.Box 132, Samara, Ethiopia

**Keywords:** COVID-19, Social welfare, Panel data analysis, Fixed effect, Random effect

## Abstract

The main objective of this study was to analyze the effect of COVID-19 on social welfare in the case of Afar regional, state, Ethiopia using panel data collected from a sample of 384 in Asyaita, Dubti Samara-Logia, and Awash town. Both descriptive statistics and econometric models were used to analyze the data. The descriptive analysis results revealed that the main source of income emanated from self-employment (81.67%), from the total households 70% of them were engaged in the service sector, due to COVID-19 the income trends of 81% of households decreased, increase expenditure on food & food items (13%) and service delivering (15%). After conducting necessary pre and post-estimation tests, the econometric model found that the three basic policy variables (number of COVID-19 victims, number of days with the COVID-19 disease and transportation ban) adversely affected the welfare of the society by lessening the income of households and growing their expenditures. Finally, considering regional experience, econometric and descriptive results, this study recommends that the government and the concerned policy maker should give more attention and subsidize the service sector, support those self-employee and daily laborers, make awareness to the society about COVID-19 epidemic, place an alternative mechanism to fill potential trade gaps.

## Introduction

The Novel Coronavirus, or COVID-19, a pandemic is a global challenge that requires coordinated efforts from governments, individuals, businesses, and various stakeholders. The pandemic causes several shocks to occur at once, including health, supply, demand, and financial shocks, causing the world economy to experience historic and unprecedented shocks [1]. There will inevitably be a general drop in economic activity both locally and internationally as a result of government efforts to contain the COVID-19 epidemic through partial and complete business closures. If the pandemic continues over an extended length of time, this contraction in economic activity results in an economic recession. Cultures with lower socioeconomic classes are more susceptible to COVID-19's rising chronic illness rates, which are exacerbated by problems with the economy and social welfare. In turn, this lowers productivity even more and drives up health care expenses, increasing poverty and, by extension, disease. A "disease-driven poverty trap" exists here [2]. From an economic standpoint, the main problem is not simply the quantity of COVID-19 instances, but also the degree of economic activity disruption, which in turn increases the level of health risks [3].

The steps people and governments take to avoid the virus will have the greatest economic impact, and this response comes from three sources [4, 5]. First, the government places restrictions on particular commercial endeavors (such as eateries, stores, etc.). Second, businesses and institutions take preventative steps like shutting down, which costs workers' income, particularly in the informal sector where there is no paid time off. Individuals also cut back on social activities like going out and traveling, which has an impact on the demand side.

our main channels: labor income, non-labor income, direct effects on consumption, and service disruption are anticipated to have an impact on poverty and inequality as a result of the rapid spread of COVID-19 in Tunisia and any potential containment measures [3]. The effects on labor income may be directly due to lost wages resulting from illness or indirect due to changes in employment and wages. Changes in remittance and public transfer patterns may have an impact on non-labor income. Consumption may be directly impacted by price increases for goods that account for a sizable portion of household budgets or by an increase in out-of-pocket medical expenses. Finally, the closure of schools and overuse of the health care system might harm welfare in the long run [6].

On March 13, 2020, Ethiopia announced the discovery of its first COVID-19 case. By October 2020, there were 96,169 confirmed cases overall, and 1469 people had died as a result of COVID-19. Along with negative consequences on health, households also experienced food and income shocks. A little more than 8.4% of households reported a job loss as a result of COVID-19 between March and October 2020. An income reduction or loss was experienced by three out of every four households. Of them, 32.3% took drastic efforts to make up for the lost income, such as selling off assets or cutting back on non-food purchases. Additionally, according to 31.4% of all homes, at least one adult member went without meals for a full day due to a lack of resources [7, 8].

COVID-19 pandemic has caused in multi-dimensional effects crosswise the Ethiopian economy. The pandemic worsened the main food insecurity and damaged the livelihood of the people in Ethiopia [9]. The Ethiopian Economic Association (EEA) projected that Ethiopia's Gross Domestic Product (GDP) will fall by 127 billion Ethiopian Birr (ETB) in the 2019/20 Fiscal Year (FY) because of the COVID-19 pandemic. According to the estimation of the EEA, the country's GDP growth will extend 0.6 percent under a severe scenario of the pandemic in 2020/2021 [8]. The pandemic heavily damage the livelihoods of the households in Ethiopia, by which the income is condensed by more than half. The subjective income measures specified that a large number of households have been bare to job loss or decreased incomes during pandemics. The negative influence of the pandemic will be severe on the welfare of exposed households. The COVID-19 pandemic is likely to have argumentative effects on agrarian households in Ethiopia. Smallholder farmers are one of the exposed groups who might be delayed from working on their land, getting into markets to sell their products, or buying seeds and other vital inputs [10]. In afar regional, state the first COVID-19 virus confirmed case was recorded on April 22, 2020. Prices of staple food and other essential supplies have significantly increased in major market centers in the Afar region, particularly in Ab’ala, yellow, and Dalifage markets, because of restrictions imposed [11].

Many studies [2, 10, 12–20] and so on a study on the macroeconomic impact of epidemic disease. Notwithstanding, most of the papers, including the above one were mainly focused on the macroeconomic effect of epidemic disease (like COVID-19), they did not give insight on the effect of COVID-19 on social and microeconomics effect (in general on social welfare), in addition, no studies addressed the regional wise social welfare effect of COVID-19. Therefore, this study was analysis the effect of COVID-19 on households' welfare in the case of the Afar regional states.

## Methodology

### Description of the study area

This study was conducted on Dubti, Samara-Logia, Asayita, and Awashi districts which were found in the Afar region.

### Type and sources of data

Both primary and secondary data were used for this study. Primary data were principally employed, which has been collected from sample representative of the society from Asayta, Dubti, A wash and Samara-loggia through questionnaires. Secondary data sources are governmental and non-governmental institutions, including both published and unpublished documents like the Afar bureau of health, Ministry of health, and online from worldometer website, WHO and other relevant information sources were used.

### Sampling technique and sample size

In this study, a two-stage sampling technique was employed. In the first stage, from all major cities of the region state, Asayta, Dubti, Awash and Samara-logo were selected by using the purposive sampling technique. The purposive selection was based on the total population, and severity of the COVID-19 virus mainly from Djibouti and Addis Ababa. In the second stage, we were simply random sampling to select the sample of the study with the help of Cochran sample size determination techniques (see Table 1).

**Table 1:** Proportion of sample households from each town of Afar regional state, 2021

Sample towns	Sampled number of population	
	Number	Percentage (%)
Asayita	96	25
Samara-Logia	115	30
Awash	96	25
Dubti	77	20
Total	384	100

1 $$\text{n}=\frac{{Z}^{2}pq}{{e}^{2}}=\frac{{\left(1.96\right)}^{2}(0.5)(0.5)}{{(0.05)}^{2}}=384.16\approx 384$$

where: is the sample size; $z$ is 1.96 to achieve 95% the level of confidence;$p$ is the proximate proportion of the population, which has the attribute in question (50% as a rule of thumb);$e$ is the tolerant marginal error as defined as in 0.05, that is, 5%maximum discrepancy between the sample and the general population.

### Method of data analysis

Descriptive statistics and econometric models were employed to achieve the objective of the study. The descriptive statistics includes means, standard deviation, minimum, maximum, frequencies and percentage and in the econometric analyses use Social Welfare Function (SWF) to estimate the effect of COVID-19 on the society's social welfare with the help of panel data analysis.

#### Model specification

The utilitarian or Benthamite social welfare function measures social welfare as the total or sum of individual incomes:

2 $$W={Y}_{1}+{Y}_{2}+{Y}_{3}+\dots +{Y}_{n}$$

3 $$W=\sum_{i=1}^{n}{Y}_{i}$$

where: $W$ is social welfare,${Y}_{i}$ Is the income of the individual,$i$ among, and $n$ is individuals in society. In this case, maximizing social welfare means maximizing the total income of the people in the society. Therefore, in this study, we proxy social welfare by the individual income to analyze the effect of COVID-19 on social welfare (on the side of income) as follows.

Fixed Effect Model

4 $${Y}_{it}={\alpha }_{i}+{\alpha }_{it}{NCV}_{it}+{\alpha }_{it}{ND}_{it}+{\alpha }_{it}{FS}_{it}+{\alpha }_{it}{EDU}_{it}+{\alpha }_{it}{ACR}_{it}+{\alpha }_{it}{TB}_{it}+{\alpha }_{it}{LT}_{it}+{\alpha }_{it}{AG}_{it}+{\alpha }_{it}{G}_{it}+{\alpha }_{it}{TSE}_{it}+{e}_{it}$$

where: ${\alpha }_{i}$ Intercept for each individual, NCV (Number of COVID-19 victim), ND (Number of days), FS (Family size), EDU (Educational level), ACR (Access to credit), TB (Transportation ban), LT (Leisure time), AG (Age), G (Gender), TSE (Types of sector of the economy), E (Error term), t (Number months).

Alternatively, Social welfare can be expressed as a function W (X1,…, Xn) of the aggregate consumption expenditure (Xi) on goods by individuals I = 1,…,n, which means that the individualistic social welfare function is also a function of individual utility levels. If there are h consumers in the economy, then an individualistic social welfare function is written *W* (*U*1,…, *Uh*), where *Uh* is the utility of *h*. The social welfare functions are also written as

5 $$W={U}_{1}+{U}_{2}+{U}_{3}+\dots +{U}_{n}$$

6 $$W=\sum_{i=1}^{n}`{U}_{i}$$

Write know, we can express SWF as a function of aggregate consumption expenditure as follows

7 $$W=\sum_{i=1}^{n}{X}_{i}$$

In such a way, we were again proxy social welfare by the individual consumption expenditure to analyze the effect COVID-19 on social welfare (on the side of expenditure) as follows.

Fixed effect model for expenditure equation

8 $${X}_{it}={\alpha }_{i}+{\alpha }_{it}{NCV}_{it}+{\alpha }_{it}{ND}_{it}+{\alpha }_{it}{FS}_{it}+{\alpha }_{it}{EDU}_{it}+{\alpha }_{it}{ACR}_{it}+{\alpha }_{it}{RLD}_{it}+{\alpha }_{it}{AG}_{it}+{\alpha }_{it}{G}_{it}+{\alpha }_{it}{TSE}_{it}+{e}_{it}$$

where ${X}_{it}$ Consumption Expenditure.

## Result and discussion

### Demographic characteristics of the sample households

The average household members that live in one house for the sample households were about 4 persons that range between 1 and 10 persons. The average age of the sample household heads was 39.13 years with maximum of 75 and a minimum of 18 years old. This showed that the mean ages of the sampled households were within the range of economically active age and they were more energetic (see Table 2).

**Table 2:** Demographic characteristics of the sample households

Variables	Mean	Standard deviation	Minimum	Maximum	Correlation coefficient
Household size	3.94	2.21	1	10	− 0.0114
Age	39.13	12.44	18	75	− 0.0356
Education level	4.58	4.25	0	15	0.2873***

Characteristics	Frequency	Percent	T/F-value		
Sex					
Female	58	15.21	0.1358		
Male	236	84.79			
Marital Status					
Married	243	63.33			
Single	90	23.33			
Divorced	51	13.33	0.0022**		
Widowed	0	0			
Total	384	100			

Source: Own computation (2021)

***, **, * indicates level of significant at 1%, 5% and 10%

The mean education level of the sample household in the study area was 4.58 ranging from 0 to first degree. The above table revealed that the mean educational levels of the sample household were very low. The correlation between the educational level of the household and the income of the households was significantly positively at a 1 percent probability level (see Table 2).

Sample respondents were composed of both male and female household heads. Out of the total sampled household head, about 84.79% were male-headed and the remaining 15.21% were female-headed households. Among the sample households in the study area majority of them (that is about 63.33%) were married, 23.33% were single, whereas 13.33% were divorced and none of them were widowed according to Table 2. F-test was employed to depict that there was an association between the marital status of the respondents and their level of income.

### Types of economic sector and sources of income

Sample respondents were engaged in three basic economic sectors, from those respondents most of them were participation in the service delivering sector, which were accounted 69.79% of the total respondents. And also sampled households participated in the agricultural and industrial sector, which were 15.83% and 14.28% of the total sample respondents, respectively. F-test was employed to depict that there was association between types of economic sector of the respondents and their level of income (see Table 3).

**Table 3:** Economic sector and source of income

Characteristics	Frequency	Percent	F-test
Source of income			
Self-employed	314	81.67	
Formal wage	115	30	
Remittance	128	33.33	1.56
Non-formal wage	138	35.83	
Rent	42	10.83	
Agricultural income	192	50.00	
Economic sector			
Agriculture	123	15.83	
Service	269	69.79	0.70**
Industry	92	14.38	

Source: Own computation (2021)

*NB* A single sample could have more that on income source and participated more than one economic sector

In the study are income sources can be broadly divided in two: agricultural income (livestock rearing and crop production and non-farm income). In general, in this study six different income sources for the households are identified, such as, farm income, non- formal wage-employment, formal wage- employment, self -employment, remittance income and rent income. This result agrees with the finding of [21]. From the total sample households 81.67% of them were received their income from self-employee and 35.83 and 33.33 of sample household also emanated their income from non-formal wage and remittance respectively. Around half of the sampled population, income generated from agriculture sector. The remaining sample households also acquired their income from formal wage and rent, which was accounted that 30% and 10.83% sample households respectively(see Table 3).

As we have seen Table 4, income trends of many sample households decreased due to COVID-19 epidemic disease, which was amounted that 81.67% of the sample household incomes were decreased because of COVID-19. From the total sample households 14.17% were increasing their income and the remaining 4.17% were does not change their income due to COVID-19. This may be due to that, most of the households were participating in service sector and self-employee source of income. According to World Bank report on 2020, the service sector was dwindled by 38% due to COVID-19 epidemic. Therefore, economic activities in the service sector and income of self-employed were highly affected by such epidemic disease compared to the other sectors and source of income. However, the income of some households was not decreased unless it was not affected or increased, this also may be the cause of, there was opportunistic entrepreneurship in the market due to COVID-19. This also creates additional income for thus opportunistic entrepreneurs. In a line with these, the income of some households also not affected by COVID-19, this was the case that, the income source of such households were from formal wage and rent, therefore such income source were not affected by such epidemic disease.

**Table 4:** Income trends of sample respondents

Variables		Frequency	Percent	F-value
Income Trends	Decrease	314	81.67	
	Constant	16	4.17	1.86
	Increase	54	14.17	
	Total	384	100	

Source: Own computation (2020)

### Expenditure related issues of the sample households

The total expenditure of the total sample households were goes to food and food related items, which were accounts for 51% of their total expenditure to food and food related items. Expenditure for service delivery like transportation, barberry, shoe shine and etc. was accounted for 12% of their total expenditure. Some sample households were not owner of some properties; therefore they must have paid some amount of money as rent, therefore, they had paid 10% their total monthly expenditure to rent. Utility expense and goods (excluding food items) accounts 6% and 7% of their total monthly expenditure of the sample households, respectively. After making expenditure to all items there might be left some amount of birr, which was as means of saving to the depositor. Expenditure for saving was 14% of total household expenditure (see Table 5).

**Table 5:** Source, percentage share and average growth rate of expenditure

Total Expenditure of sample households/individuals in Birr	Total expenditure amount in birr (ETB)				Average growth rate of total expenditure	Average percentage share of expenditure from the total income
	March 13	April 13	May 13	June 13		
Food and food related items	268,368	295,205	336,533	390,379	13.3%	51%
Service	63,145	68,828	81,217	97,461	15.7%	12%
Utilities	36,835	36,835	36,835	36,835	0.0%	7%
Rent	52,621	52,621	52,621	52,621	0.0%	10%
Saving	73,670	67,776	59,643	51,889	− 11.7%	14%
Goods( exclude food items)	31,573	33,152	36,798	41,582	9.7%	6%
Total	526,212	554,417	603,647	670,767		100%

Source: Own computation (2021)

COVID-19 epidemic has its own effect on social, economic and psychological in the world generally and in Ethiopia Afar region particularly. From the finding of our study from March 13, 2021 to June 13, 2021 the overall expenditure of households had significant changes, except expenditure on utilities and rent. From the above table, food and food related items and service expenditure were relatively significant increase after the COVID-19 epidemic. The total expenditure on food and food related items were increased by 13.3% per month on average after COVID-19 epidemic. This may be due to, difficulty in distribution and transportation of food and food related goods and decline in production capabilities (see Table 5).

Total expenditure on the side of service delivering activities was increased by 15.7% on average per month. Which was witnessed by specifically increased the cost of transportation. Expenditure on goods (excluding food related items) like expenditure on cloth, shoe, kitchen materials, construction materials and etc. had increased by 9.7% on average per month, this was also due to the problems of distribution and transportation of such products(transportation ban). Notwithstanding, sample household expenditure on rent (like expenditure properties rent) and utilities (like expenditure mobile air time and internet packages, water and electricity bill) hadn’t a significant effect due to COVID-19 epidemic. Finally the saving behaviors of households were hurt by such disease, which was decreased by 11.7% per month on average from the total household expenditure for saving. In general, due to COVID-19 epidemic disease expenditure on food and food related items and expenditure on service were increased significantly relative to other item of expenditure, plus more than half of total expenditure was accounted for food and food related items. Therefore, the wellbeing as well as the welfare of sample households was highly affected by COVID-19 epidemic disease (see Table 5).

### Effect of COVID-19 on income and consumption expenditure

The analysis done using Stata 14 and E-views 9 is as presented below. During estimation, we had to made necessary pre estimation test (stationary and houseman) and post estimation test (heteroscedasticity, serial autocorrelation, cross sectional dependence, multicollinearity and endogeneity). The objective of the study was analysis the effect of COVID-19 on social welfare of afar regional state. Linear panel data analysis was used to investigate the effect of COVID-19 on social welfare. When we had been analyzed the effect of COVID-19 on social welfare, by categorized the explanatory variables as a policy variables (number of COVID victims, number of days, transportation ban, types of economic sector) and control variables(leisure time, gender, access to credit, family size and educational level)(see Table 6). Accordingly, variables assumed to have influence on social welfare in different contexts were tested in the model and seven out of nine variables were found to be significant in income equation and five out of eight variables also to be significant in expenditure equation. Among variables fitted into the income fixed effect model:- number of COVID-19 victims, number of days, transportation ban, leisure time, gender, types of sector of the economy, access to credit and family size significant. On the side of expenditure fixed effect model: number of COVID-19 victims, number of days, transportation ban, gender and access to credit significant.

**Table 6:** Fixed effect estimation result for income and consumption expenditure

Fixed effect model for income equation			Fixed effect model for expenditure equation		
Variables	Coefficient	Standard Error	Variables	Coefficient	Standard error
income			expenditure		
Number of COVID victims	− 0.270***	0.070	Number of COVID victims	0.285*	0.29
Number of days	− 0.159*	− 0.080	Number of days	0.541***	0.05
Transportation ban	− 0.86**	0.41	Transportation ban	0.1802**	0.18
Leisure time	− 0.18**	0.06			
Gender	− 0.05**	− 0.002	Gender	0.325**	0.33
Types of economic sector	− 0.011***	0.003	Types of economic sector	− 0.521	− 0.52
Access to credit	0.131*	− 1.868	Access to credit	0.8925*	0.89
Family size	− 0.987	− 19.738	Family size	0.45875	0.46
Education level	0.241	12.044	Education level	256.221	256.22
Constant	0.725	13.426	Constant	2889.51	2889.51

Source: Model output (2021)

***, **, * refers to 1%, 5% and 10% level of significance, respectively

#### Number of COVID-19 victims

The model reveals that number of COVID-19 victim has a significantly positive relationship with income of households and expenditure at 5% and 10% probability level, respectively. As the number of COVID -19 victims by one person, the income of households decreased by 0.27% and the expenditure of households also increased by 0.28% per month (see Table 6). This may be due to the fact that, as the number of COVID-19 victims increase the government takes a measurement to restrict the movement of labor, which makes decreasing labor additional income and ban the distribution of goods and service which diminishing the quantity supplied and increase price of the product, in addition the peoples may be frustrated and not willing to move place to place and work. Therefore, these factors may have its own contribution to decrease the income of households and increase the amount of household expenditure. This results is similar with.

#### Number of days

The result also showed as there is a positive relationship between expenditure and number of days starting from March^1^ 13, 2020 and negative relationship between income of households and number of days starting from March 13, 2020 at 1% and 10% probability level respectively (see Table 6). When the number of days increased by one more days with COVID-19 epidemic, the income level was decrease by 0.15% and expenditure of households was increased by 0.54% per month. This may be due to, if the number of days with COVID-19 epidemic increase, there were a caused for shrinkage of both intra and inter regional trade, investment, tourism, manufacturing sector and a little bit decreasing in agricultural sector production. Such factors had their own contribution to decrease the income of households and increase the amount of household expenditure. This results is supported [22, 23].

#### Transportation ban

This variable affects income negatively and expenditure positively in significantly at 10% probability level. Thus, keeping other thing remain constant; the probability of the level of income decreased by 0.86% and expenditure increased by 0.18% when there was transportation ban compared to there was not transportation ban. As we have seen before,from the total sampled households 81.67% and 35.83% of their income were emanated from self-employee and non-formal (daily labor) wage, in such a way such activities required moving place to place to work (see Table 6). However, due to transportation ban it was difficult to do such activities as usual; therefore, at the end of the day the income of households will be decreased. On the side of expenditure, it increased due to transportation ban example between Dubti, Logia and samara town, the initial payment is increased by hundred percent this indicates the expenditure of households leads to increased [2, 24–26].

#### Leisure time

High wages due to working very long hours diminishes economic welfare. Leisure has quantified by hour and it has economic value on increase social welfare and/or the remaining hour from 160 h per month. The result also showed as there are negative relationships between of leisure time and income of household at 5% probability level (see Table 6). This indicated that, when the household leisure time increased by one hour, the level of household income was decreased by 0.18% per month, however their welfare may be improved because of additional leisure time. The reason behind the screen was due to, as postulated on the above total sampled households 81.67% and 35.83% of their income were emanated from self-employee and non-formal (daily labor) wage, therefore as they did not work as usual or takes more leisure time their level of income was decreased. This finding is similar with [6, 27]

#### Gender

It was found that male headship has a negative and significant effect on income of households at 5 percent probability level. Thus, keeping other thing remain constant; the probability of level of income decreased by 0.05% and the expenditure of the households increased by 0.325% per month when the household head is male (male headed households) compared to female (see Table 6). This will be the cause for, most of income was generated by male rather than female household head, and therefore any factor affected on income were initially affected male headed household rather than the female. In addition, male households head were diversified their income sources than female household head, this meant by male household participated in different non-formal income generating activities [21]. However, those non-formal or self-employee income sources were affected by COVID-19 epidemic. Finally most male households was spent more money than female, therefore the expenditure of household was increased when the household head male. This result is supported by [2, 28]

#### Types of economic sector

As expected, a type of economic sector was significant at 1% probability level, and has a negative relationship with the level of income of households. As the economic sector was service sector the probability of households income decreased by 0.011% compared to the industry and the agricultural sectors (see Table 6). As we have seen before around 70% of sample households was engaged in service sectors. The earliest and largest impacts are more likely to occur in the service sector, such as transport, retail sales, entertainment, tourism, and personal services, including those engaged in the gig economy, rather than in agriculture, large manufacturing, and public, professional, ICT, and financial services. In addition according to World Bank report on 2020, the service sector was decreased by 38% due to COVID-19 epidemic disease. This finding is similar with [25, 26, 29–31]

#### Access to credit

Access to credit affect the level of income of household’s and expenditure households positively and significant at 1 percent level of significance. The result defines to our prior expectation. This means credit utilization by household would increase income level by 0.13% and expenditure by 0.8925% (see Table 6). This means, when households got an additional credit, the level of an additional income was increase, this leads to increase the additional expenditure.

Generally, as we have seen in the above finding due to COVID-19 epidemic disease, the expenditure of the households increased significantly with trade off decreasing in income of the sample households. This finding is similar with [30, 32, 33]

## Conclusions and policy implications

This study attempts to investigate effect of COVID-19 on social welfare and trade in case of Afar Regional State using the sample data collected from 384 randomly selected households from three Samara-logia, Asyaita, Dubti and Awash towns, Afar region Ethiopia. Both descriptive analysis and econometric estimation (panel data analysis) results have been used to address the objective of the study. The descriptive statistics revealed that around 70% of sampled household’s source of income was served in service delivering sector of the economy and the remaining 16% and 14% of households were employed in agricultural and industry sectors respectively. The main source of income of households were generated from self-employee, agriculture income and non-formal wage, accounted for 81.67%, 50% and 35.83% of total income were stemmed from those source respectively.

Due to COVID-19 disease 81% of household’s income was decreased, 14% of household’s income increased and 5% of household’s income remain the same after such disease. Total expenditure items of sampled households were changed after COVID-19 i.e. expenditure for food & food items and service delivering were significantly increased compared to the remaining expenditure items, which were constitutes around 13% and 15% increment per months. The control variables of panel data model also its own significant effect on welfare i.e. leisure time and sex of households head had negative and access to credit has positive and significant effect on income of households, on the expenditure side of the household gender and access to credit had positive and significant effect on it. Considering regional experience, econometric and descriptive results, this study recommends that the government and the concerned policy maker could undertake the following policy actions for adversative effect of COVID-19 on social welfare. The government should give more attention to service sector (to protect the rate of unemployment) and supported those self-employee and daily laborer in a means of financial and material needs, responsible bodies should made encourage investment on food and food items production, processing and distribution specially in the regional state like tax free, provided land, electricity, water, agricultural inputs, access to credit and as long as public transportation to the society; the government and responsible bodies should makes awareness, provide mitigation and health materials kits, prepared well organized quarantines to keep the spread of the disease, the health status of the victims and increased the numbers of cure. In addition, the regional government should not ban all in all aspect of the transportation, especially the distributing and trade of food and foods items, place alternative mechanism to fill potential trade gaps.

Mobilize social self-help institutions and them with the formal structure to provide a coordinated support to the most vulnerable population during the pandemic. Put in place alternative mechanism to fill a potential import deficit, these may include planting short-season and early-maturing crop varieties, and prioritizing irrigation schemes for selected foods crops (e.g. potato, maize, etc.), With immediate effect, put in place measures that will ensure uninterrupted supplies of (chemical fertilizers, improved seeds, pesticides and herbicides as well as livestock medicine), these will minimize the adverse effects of the pandemic in the agricultural sector, initiate discussions with commercial banks on rescheduling bank loan repayments and write off interest payments for severely affected sectors until the shock is abated.

The National Bank of Ethiopia needs to consider reserve rate relaxation to enhance banking liquidity, the National Bank of Ethiopia shall initiate discussions to reduce interest rates to stimulate the economy, initiate discussions with financial institutions to support exporters by increasing foreign trade credits, deferring loan payments and extend debt rollovers.

## Data Availability

Data will be made available on reasonable request of corresponding author.

## Footnotes

1 Federal ministry of health Ethiopia has confirmed first coronavirus disease (COVID-19) case.
